# Electrical and Optical Characterization of SAW Sensors Coated with Parylene C and Their Analysis Using the Coupling-of-Modes (COM) Theory

**DOI:** 10.3390/s22228611

**Published:** 2022-11-08

**Authors:** Nikolay Smagin, Meddy Vanotti, Marc Duquennoy, Lionel Rousseau, Hassan Alhousseini, Virginie Blondeau-Patissier, Mohammadi Ouaftouh, Laurie Valbin, Etienne Herth

**Affiliations:** 1 IEMN (Institut d’Électronique de Microélectronique et de Nanotechnologie), UMR CNRS 8520, Univ. Polytechnique Hauts-de-France, CNRS, Univ. Lille, 59313 Valenciennes, France; 2 FEMTO-ST (Franche-Comté Électronique Mécanique Thermique et Optique—Sciences et Technologies), Département Temps-Fréquence, UMR CNRS 6174, Université Bourgogne Franche-Comté, 26 Chemin de l’Épitaphe, 25030 Besançon, France; 3 ESYCOM Lab UMR, CNRS 9007, ESIEE-Paris, Univ. Gustave Eiffel, 77454 Marne-la-Vallée, France; 4 Centre de Nanosciences et de Nanotechnologies, CNRS UMR 9001, Université Paris-Saclay, C2N-Palaiseau, 10 Boulevard Thomas Gobert, 91120 Palaiseau, France

**Keywords:** surface acoustic wave (SAW), biocompatible polymer, coupling-of-modes theory, sensing layer, chemical SAW sensor, laser Doppler vibrometry

## Abstract

In this paper, we present how complementary characterization techniques, such as electrical measurements with a vector network analyzer (VNA), optical measurements with a laser Doppler vibrometer (LDV), and numerical simulations with the finite element method, coupled with spectral domain analysis (FEMSDA), allow us to independently access different properties of a SAW device and fully characterize its operation using the coupling-of-modes theory (COM). A set of chemical SAW sensors coated with parylene C layers of different thicknesses (1, 1.5, and 2 µm) and an uncoated sensor were used as test samples. The sensors represent dual-channel electroacoustic delay lines operating in the vicinity of 77 MHz. The IDTs consist of split aluminum electrodes deposited on a AT-cut quartz substrate. The thickness-dependent influence of the parylene C layer was observed on the operating frequency (SAW velocity), static capacitance, attenuation, crosstalk, and reflection coefficient. COM parameters were reported for the four cases considered; measured and simulated data show good agreement. The presented approach is suitable for the design, characterization, and validation of polymer film-coated SAW sensors.

## 1. Introduction

Biocompatible electronic devices [1] and sensors [2,3] are rapidly developing fields of technology that could provide pathways to green, diagnostic, and environmentally friendly electronics [4]. So far, a large number of biomedical micro- and nano-devices, such as neural probes and retinal prostheses, have required packaging materials made of poly-para-xylenes, also known as parylene [5,6,7]. Parylene has excellent barrier properties, is insensitive to solvents, is hydrophobic and biocompatible, and has a low coefficient of friction (static and dynamic friction coefficients are the same). It is used in the medical industry to make medical devices biocompatible, in electronics for electrical insulation, in mechanics for protection against corrosion and pollution, and as a dry lubricant [8]. Parylene C, the monochloro-substituted compound, is the most commonly used [9]. Among the biocompatible parylene, parylene C is very suitable for piezoelectric technology, which includes applications in sensing [10], actuation [11,12], and energy harvesting [13]. Of particular interest is the study of chemical sensing using surface acoustic waves (SAW) [14,15], based on the principle that some physical parameters are associated with a chemical agent’s presence. The latter can cause a perturbation of the propagation velocity SAW, which in turn can be monitored by velocity measurements or changes in frequency, phase, or amplitude of an electrical circuit using a SAW device [16]. SAW devices coated with thin, sensitive polymer films are known for detecting small amounts of various chemical gazes in the ambient atmosphere [17,18].

Two important questions arise in the development of a SAW sensor coated with a viscoelastic polymer. First, the operation of the sensor in terms of quality factors and losses must remain unchanged to some degree in order to perform measurements with a circulator [14] or other peripheral circuitry. Second, the thickness of the coating must be chosen to achieve the desired gas sensitivity and selectivity [19]. The existence of an optimal film thickness that yields maximum gas probing sensitivity is attributed to the gas sorption specifics of the sensing layer [20,21]. The above illustrates the importance of analyzing and modeling sensor systems based on SAW [22] to gain insight into the effects of design parameters and polymer coating on sensor performance. Among available modeling methods, such as the finite element method and perturbation theory [23,24,25], the coupling-of-modes (COM) theory is of interest as it enables a rapid SAW sensor simulation and optimization using numerous test configurations [26]. In this phenomenological model, it is necessary to introduce COM parameters that are determined independently elsewhere.

This paper presents experimental results of the characterization of a series of two-channel sensors based on SAW delay lines covered with parylene C sensing layers of different thicknesses. It is shown how a combination of electrical measurements (using a vector network analyzer) and optical measurements (using a laser Doppler vibrometer) [27,28,29] can be used to determine parameters describing the effect of the viscoelastic polymer coating. These parameters are frequency shift, SAW attenuation, velocity, reflection coefficient, and static capacitance. The measurement results obtained with the two methods show agreement and complementarity. Measured values can be used directly in the COM model of the SAW device to simulate the presence of the parylene C coating [26]. The reported approach is applicable for validating simulation results and optimizing sensor performances. It is first applied to the COM model formalism, but since it deals with natural physical properties, it can also be useful for sensor development using other mentioned simulation methods.

## 2. Test Samples and Experimental Setup

The fundamental constituting element of a SAW device is an interdigital transducer (IDT) deposited on a piezoelectric substrate [30]. An IDT consists of two comb-shaped thin metal electrodes forming interdigitated fingers with an r overlap distance of *W* [20,21,22]. In the simplest case, the electrode finger’s periodicity is constant and denoted as *p*. The propagation velocity of the elastic surface waves in the piezoelectric substrate is referred to as *v*. It depends on the type and (if applicable) the crystal orientation of the substrate.

When an electrical voltage is applied between the two adjacent electrodes, an accumulation of charges with alternating signs is produced. The electric field between each pair of fingers induces mechanical displacements due to the reverse piezoelectric effect. If the applied AC voltage has a frequency $f_{0}=v/2p$, mechanical vibrations are added constructively, resulting in two surface acoustic waves propagating in opposite directions. The wavelength of the generated SAW is double the electrode periodicity: λ=2p. The described emitting mechanism corresponds to the elementary bidirectional IDT of the single-electrode type. A variety of more sophisticated IDT electrode configurations allows for unidirectional emission, or, for example, reducing acoustic reflections from the electrodes by splitting them [31].

The direct piezoelectric effect allows using another IDT as a receiver by placing it in front of the emitting IDT. Since the propagation velocity of elastic waves is five orders of magnitude lower than that of electromagnetic waves, such a device forms a very efficient radio frequency delay line. In addition, acoustic waves can easily interact with the propagation environment, which enables the conversion of the delay line into a SAW sensor capable of measuring the environmental physicochemical properties. Specific sensitive layers placed in the propagation region of surface acoustic waves could further improve detection capabilities. For example, such layers can absorb the target chemical agents, resulting in additional mass loading during SAW propagation. This type of sensing relates to the gravimetric SAW sensors [32].

A total of four parylene C-coated chemical SAW sensors were tested: an uncoated one and the others coated with 1, 1.5, and 2 µm-thick films. The conformal parylene coating was applied by a vapor deposition process under medium vacuum conditions and at room temperature.

The AT-cut (YXl/$-35.25^{{}^{\circ}}$) quartz was chosen as a substrate because of its first-order temperature coefficient (TCF) close to zero [33], and the Rayleigh wave propagation mode providing high sensitivity in gaseous media [17]. Rayleigh SAW propagation has the added advantage of allowing optical measurements of the out-of-plane displacement component with a laser Doppler vibrometer (LDV).

**Figure 1:**
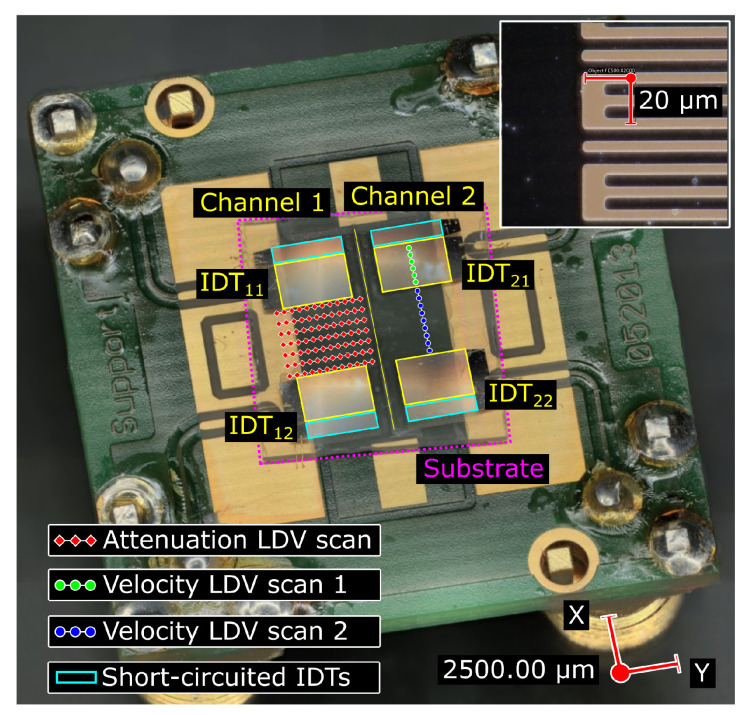
Microscopic image of the two-channel chemical sensor. The substrate tilt (magenta) and LDV scan regions (red, green, and blue markers) are visible. The positions of the markers do not correspond to the exact scanning points. The split electrode structure is depicted in the inset in the top right corner.

Each sensor consists of a dual-channel delay line (labeled ’Channel 1’ and ’Channel 2’ in Figure 1) placed on a tilted substrate. The device is intended for incorporation into a two-channel oscillator circuit [14,34] where one line is used for measuring and the other as a reference. Each SAW interdigital transducer consists of 52 split-finger electrode pairs with a grating period of 10 µm and a thickness of 200 nm. The electrode fingers are made of aluminum (200 nm thick) and are realized in split configurations to minimize SAW reflections (see the inset in Figure 1). The wavelength of 40 µm results in an operating frequency of nearly 78 MHz as the wave speed approaches 3150 m/s [33]. The total length of the IDT is 2.08 mm, the length of the non-metalized zone between the emitter and the receiver is 3.2 mm (80 wavelengths), and the aperture size is 3 mm, which corresponds to 75 wavelengths. On the back side of each emitter/receiver IDT, a second short-circuit IDT is placed (20.5 pairs of electrodes, shown with cyan outlines in Figure 1). For more details, see [35].

In the next sections, the reported electrical S-parameter measurements were made using a Tektronix TTR 503A Vector Network Analyzer (VNA); impedance characteristics were obtained using an HP 4195A VNA coupled to an HP 41951-61001 impedance meter (Section 3). Optical characterization, including SAW attenuation measurements (Section 4), velocity measurements (Section 5), and SAW propagation mapping (Section 7), were carried out with a Polytec UHF-120 scanning LDV [36].

## 3. Electrical Sensor Characterization

The electrical measurements are able to directly reveal the effects of the parylene film coating on the operation of the sensor. The two obvious consequences of the layer deposition are increased insertion loss and a downward frequency shift. Both are due to the mass loading of the piezoelectric substrate and the viscoelastic absorption in the film. The third potentially disturbing effect is the influence of the dielectric constant of the polymer, which modifies the static capacitance of IDTs [26]. Indeed, parylene C dielectric constant $\varepsilon_{p}$ is 2.95 [15], and for AT-quartz $\varepsilon_{p}=5.506$ [31].

**Figure 2:**
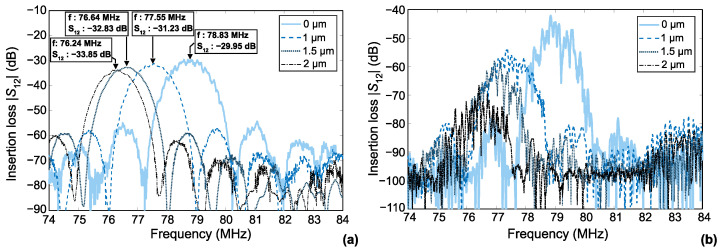
S${}_{12}$ measurements: (**a**) between opposite IDTs of the same channel (IDT${}_{11}$ and IDT${}_{12}$ in Figure 1); (**b**) opposite IDTs belonging to adjacent sensor channels (‘IDT${}_{11}$’ and ‘IDT${}_{22}$’ in Figure 1).

All $S_{12}$ measurements reported in Figure 2a were performed at 22 °C ambient temperatures. First, in the case of the naked sensor (referred to as 0 µm in the figure), the use of a weak piezoelectric substrate such as AT-quartz (electromechanical coupling $K^{2}=0.00155$, [31]) with a relatively small number of IDT finger pairs results in a rather high value of insertion loss of about −30 dB. No $S_{12}$ ripples are observed in the passband region due to a triple-transit echo and/or reflection from the electrode grid of the receiving IDT [31]. When the polymer coating is added, the insertion loss increases progressively with thickness: it starts at −31.85 dB for a coating thickness of 1 µm and ends at −33.7 dB for the 2 µm thick layer. In the most pronounced case, the increase in insertion loss is 3.7 dB compared to the uncovered condition. This rather moderate added loss does not significantly affect the operation of the SAW sensor.

Similarly, the initial value of the center frequency (78.82 MHz for the uncoated sensor) shifts with increasing parylene coating thickness. For the 2 µm coating, the center frequency is 76.2 MHz, which corresponds to a relative variation of 3.3%. Table 1 shows the absolute and relative (compared to the uncoated state) values of insertion loss and frequency shift.

In [35], LDV measurements showed that substrate tilt leads to an interchannel crosstalk (Figure 2b). Crosstalk decreases the phase stability of the circulator circuit. The obtained crosstalk value for the uncovered sensor was −15.2 dB. In the case of the polymer-covered sensors, the $S_{12}$ measurement between opposite transducers belonging to different channels (‘IDT${}_{11}$’ and ‘IDT${}_{22}$’ in Figure 1) also shows the presence of crosstalk between the channels (Figure 2b). Here, the crosstalk values are estimated as the differences of the $S_{12}$ values obtained for the same and adjacent channels, as listed in Table 1. The presence of a coating layer leads to a crosstalk decrease: it is −23.4 dB for the 1 µm layer and decreases to −33.4 dB for the 2 µm layer. The noise-like profiles in Figure 2b are always at least 20 dB above the VNA noise level, which is −100 dB. Their “spiky” appearance is due to multiple echo returns traveling between the two sensor channels. An additional acoustic absorber between the channels is able to solve this problem but increases the complexity of the device manufacturing. The mechanisms of crosstalk are analyzed in detail in Section 7 on the basis of optical measurements.

**Figure 3:**
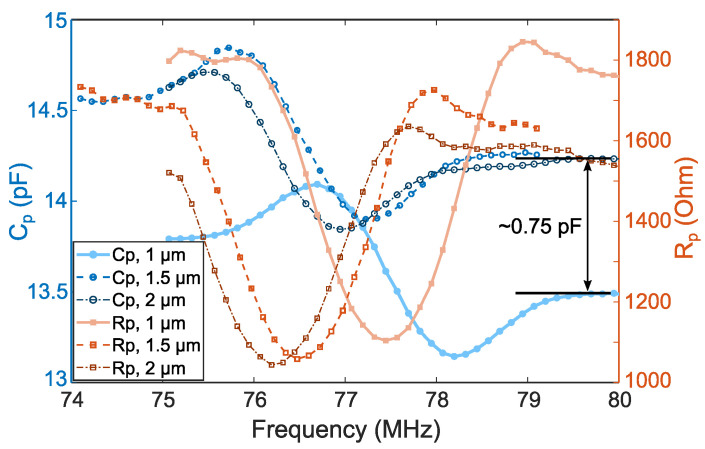
$C_{p}$ (right axis and circle markers) and $R_{p}$ (left axis and square markers) equivalent circuit parameters measurement with an impedance meter HP 4195A.

It is known that the additional static capacitance when mounting a SAW sensor in a circulator can cause additional frequency shifts, signal attenuation, and increased sensitivity to crosstalk [37,38]. Figure 3 illustrates the impedance modification in terms of capacitance $C_{p}$ and resistance $R_{p}$ for a parallel equivalent circuit. It should be mentioned that the measurement for the uncovered sensor was not possible as all three available sensors were already covered with parylene C at this stage of the study.

The central frequency shifts seen in Figure 3 correspond to those found in the insertion loss measurements in Figure 2a. The $C_{p}$ variation between sensors coated with 1.5 and 2 µm film is almost negligible. However, the discrepancy with the 1 µm parylene-coated sensor is approximately 0.75 pF, which corresponds to a 5.5% relative variation.

The measured values of insertion loss, frequency shift, and static capacitance varied within the limits of several percent, which is tolerable for a correct sensor operation. However, having only electrical measurements is not sufficient for the sensor simulation. The next section is dedicated to supplementary optical characterization of the sensors, providing a more detailed vision of the modification of sensor operations due to polymer coating.

## 4. Optical Attenuation Measurement with LDV

Optical measurements of attenuation might be insightful as they allow acoustic mechanisms as diffraction and propagation losses to be distinguished from other possible non-acoustic sources of loss, such as modifications of the electromechanical coupling, transduction coefficient, or other second-order effects.

To obtain data on the energy distribution in the acoustic beam, complete cross-sectional amplitude profiles were acquired in the propagation region between the emitting and receiving transducers. The location of the scan profiles is shown by red diamonds in Figure 1. Each was formed by 245 scanning points spaced by Δy=15.2 µm. In total, there were 7 profiles separated by Δx=0.5 mm distance. Thus, 123% of the 3 mm IDT aperture was covered by the 3.7 mm sectional scan.

The emitting IDTs were excited with 52 cycles of tone bursts provided by an arbitrary waveform generator (Tektronix AWG 7051A). The excitation frequency corresponded to the values given in Table 1. The amplitude of the electrical signal increased to the 20 V${}_{0-p}$ level using a RF power amplifier (Amplifier Research 50W1000A, 50 Watts). Optical signal acquisition was performed at an average rate of 128 sweeps.

**Figure 4:**
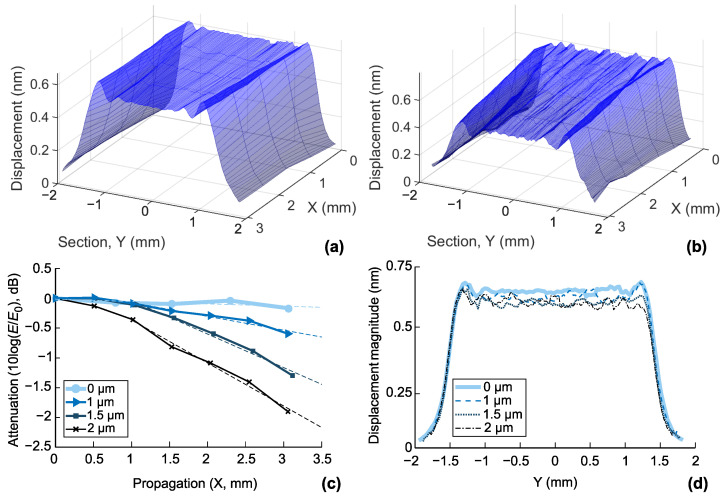
Measurement of SAW attenuation with LDV: (**a**,**b**) 3D profiles of SAW displacement cross-section distribution for the non-covered and the sensor covered with 2 µm thick parylene layer, respectively; (**c**) profile of energy decaying during propagation; (**d**) energy distribution at the emitting point (corresponding to *X* = 0 mm in (**a**,**b**)).

Three-dimensional profiles of SAW displacement cross-sectional distribution are presented in Figure 4 for non-covered (a) and 2 µm covered (b) sensors. One can observe that a wide aperture W=75λ assures a near-field operation in both cases (no diffraction is observed). Increased attenuation and profile perturbation due to surface irregularities are clearly observable for the covered sensor (Figure 4b).

The attenuation value can be found by calculating acoustic energy decay in sectional profiles distributed over the distance between the emitter and the receiver. For this purpose, the energy *E* for each section was calculated by integrating square amplitude values. Figure 4c represents energy evaluation in logarithmic form, where $E_{0}$ stands for the energy of the first section closest to the emitting IDT. The linear fitting of the energy decay gives the value of attenuation in dB/mm (Figure 4c); the obtained values are cited in Table 2. The first column corresponds to optical measurements, while the second one contains the electrical measurements converted in dB/mm. For this conversion, the midpoint-to-midpoint distance between the emitting-receiving IDT centers (5.2 mm) was taken as a reference, and the values of insertion loss increase from the 5th column of Table 2 were used. The value of attenuation due to free space propagation (0.04 dB/mm) was added to the final values.

Thomas et al reported the attenuation value of ST-cut quartz at 78.8 MHz from electrical measurements to be about 0.036 dB/mm, which is close to our optically found value of 0.04 dB/mm [39]. It is worth mentioning that the attenuations at these relatively low microwave frequencies are low, resulting in relatively low measurement accuracies. Indeed, the data reported in various publications on SAW attenuation at frequencies below 100 MHz have significant discrepancies. For example, the approximate equation from the less recent work [33] gives a value of 0.016 dB/mm for the same case of 78.8 MHz SAW at ST quartz.

At higher attenuation values, the VNA data converge to the results of the optical LDV measurements. Comparing the first two columns of Table 2, we can see the values from electrical measurements are systematically higher than those found by optical measurements. This discrepancy is about 0.05 dB/mm on average, which can be attributed to the influence of connection wires and packaging.

The eventual change in the value of the transduction efficiency can also be optically assessed. It is considered a second-order effect compared with attenuation [26]. The SAW displacement profiles near the emitting IDT confirm this statement (Figure 4d). Here, the observed decrease in energy does not exceed the rates due to the attenuation quantified in Table 2.

## 5. Sound Velocity Measurements

LDV measurements are able to estimate the relationship between the SAW velocity variations due to the thin film layer coating and the frequency shift observed with electrical measurements in Section 3. As the electrode grating is susceptible to having an additional impact on the sound velocity [31], the measurements were performed both in the IDT zone and in the free space between the receiving and emitting IDTs. The corresponding measurement regions are shown in green (’LDV scan 1’) and blue (’LDV scan 2’) circles in Figure 1. The ’IDT${}_{22}$’ transducer was used as an emitter.

Several robust methods are applicable to measure SAW velocity accurately, for example, a wavelet transform [40] or a two-dimensional fast Fourier transform applied to signals recorded at uniformly spaced distances [41].

In the present study, velocity values were obtained by performing 1D-scanning in the SAW propagation direction and applying the slant-stack transformation, also known as the *p*-ω transformation or oblique summation [42]. In terms of precision, this method is as good as the others (2DFFT and wavelet). However, it is suitable, in particular, for the case of Rayleigh waves [43,44] and allows the phase velocity to be determined directly as a function of frequency.

The sound speed measurements were performed following the guidelines from [45] regarding the scan point spacing and the total number of points. The spatial scan step Δx = 9.55 µm and the total scan points were 295 and 325 for the IDT and the free space zone scanning, respectively. These settings allowed obtaining the measurement precision of ±0.5 m/s. The fourth and fifth columns of Table 3 contain measurement results for the two considered zones. One can observe a high degree of agreement between the VNA operating frequency measurements (second column) and the frequency values recalculated from the values for the IDT region (sixth column).

The discrepancy between the sound velocity values corresponding to the free space and the IDT zone exceeds the measurement uncertainty and increases with the increasing parylene layer thickness. Due to the electric and additional mass loadings, the sound velocity is lower in the metallic IDT grating zone [31,46]. The use of thin (h=200 nm) aluminum electrodes at very-high frequency ranges (30 to 300 MHz) minimizes the mass loading factor because the volume density of aluminum and the *h* to period *p* ratio are small (p=0.5 of wavelength). In the considered case, h/p=1%.

Electrical loading is caused by the fact that the conductive electrodes on the piezoelectric surface short-circuit the electric fields, reducing the energy flow in the wave and, therefore, the velocity [47]. The AT-quartz is a so-called “weak piezoelectric material,” so the electric loading is relatively low. This term is almost constant and is approximately proportional to the piezoelectric coupling coefficient ($K^{2}$ = 0.00155 in the considered case [31]).

However, even under these soft conditions imposed by the IDT on the piezoelectric substrate, the difference in the sound velocity values between the IDT and the free space zones is detectable. As observed, in the case of the 2 µm coated sensor, the frequency shift in the free space region is 3% (76.42 MHz) instead of the 3.25% observed experimentally. This difference in sound velocity in the two regions cannot be directly accessed by VNA measurements. However, the free space sound affects the group delay value, and these two sound speed values are useful for verifying the numerical simulations of polymer-coated SAW sensors [26,31].

It is worth mentioning that, with the increase of IDT operating frequency up to the ultra-high frequency range (300 to 3000 MHz), the ratio of electrode thickness to period increases (up to 6% [21]), making the influence of mass loading more pronounced. The increased mass loading of the electrode will also occur when a denser metal is used in electrodes, such as platinum.

## 6. Validation of Experimental Data for COM Model Analysis

### 6.1. Modification to COM Parameters Due to Polymer Coating

A comprehensive presentation of the COM model can be found in numerous sources [26,31,47]. COM equations allow describing such phenomena as the electric excitation, propagation, and mutual reflections of counter-propagating, plane-wave-like acoustic modes in a 1D effective continuous medium, as well as the generation of electric currents [47]. The parameters used in the model are (i) SAW propagation velocity in electrodes grating region *v*, (ii) SAW attenuation coefficient γ, (iii) electrode reflectivity κ, (iv) transduction coefficient α, and (v) static capacitance per interdigital transducer (IDT) unit wavelength $C_{p}$.

A surface modification due to polymer coating changes the SAW propagation velocity *v* and attenuation γ, leading to a change in the initial SAW complex propagation factor $\beta_{0}=k-j\gamma$, where *k* is the wave number. One introduces the perturbed propagation factor $\beta_{p}=\beta_{0}+\Delta \beta$, as follows [23,24]:

(1) $$\Delta \beta =-\frac{\Delta v}{v}-jk\frac{\Delta \gamma }{k},$$

where Δ*v*/*v* and Δγ/k represent the normalized SAW velocity and attenuation modification resulting from the polymer coating. This perturbation modifies the COM dispersion relation and affects all P-matrices describing the SAW device.

Additionally, it is necessary to account for changes in the static capacitance per IDT unit length defined as $C_{p}=C\lambda_{0}$, where *C* is total IDT capacitance and $\lambda_{0}$ is the operating wavelength. In practice, one often uses the normalized capacitance $C_{n}=C_{p}⁄W$, where *W* is the IDT aperture. The addition of a dielectric medium between the electrode fingers of an IDT induces an extra capacitance C${}_{n1}$ [38]:

(2) $$C_{n1}=\varepsilon_{0}\varepsilon_{1}\left(\frac{h}{a}\right),$$

where $\varepsilon_{1}$ is the relative dielectric constant of the coating (=2.95), $\varepsilon_{0}$ is the vacuum permittivity (8.854187.10${}^{-12}$ F/m), and *a* is IDT electrode width. Thus, the modified IDT capacitance $C_{n}^{{}^{\prime }}=C_{n}+C_{n1}$. This simplified formula predicts an addition of 0.15 pF/µm to the initial normalized IDT capacitance for the sensors considered here.

### 6.2. Extraction of COM Parameters from Measurements and Numerical Simulations

**Figure 5:**
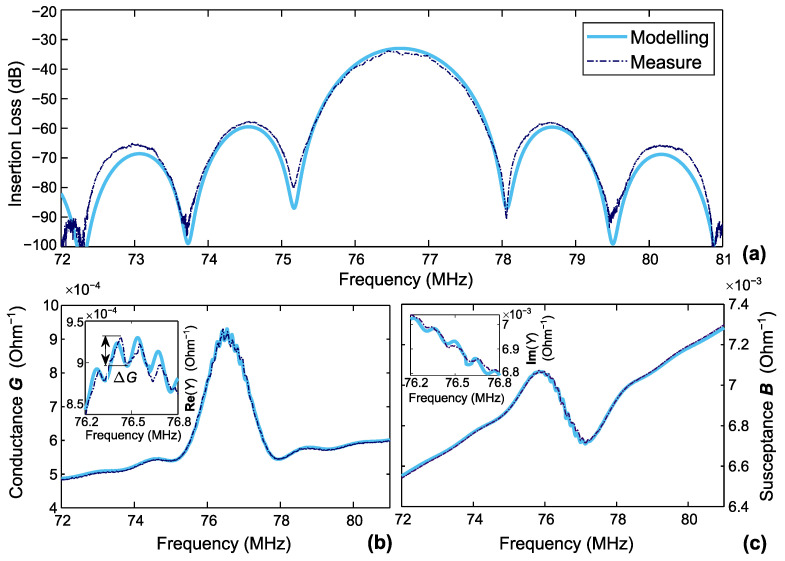
Simulated (solid lines) and measured (dash-dot lines) electric response for 1.5 µm Parylene-covered sensor: (**a**) insertion loss; (**b**) conductance; (**c**) susceptance.

COM parameters for the four tested sensors are presented in Table 4 in the normalized form [47]. Thus, $\kappa_{p}=\kappa \lambda_{0}$ is the reflection coefficient per IDT wavelength $\lambda_{0}$. The normalized transduction coefficient is as follows:

(3) $$\alpha_{n}=\alpha_{p}/\sqrt{\frac{W}{\lambda_{0}}},$$

where $\alpha_{p}=\alpha \lambda_{0}$ is the transduction per unit length. Attenuation per unit wavelength is given as $\gamma_{p}=\gamma \lambda_{0}$. As an example, these values have been used to simulate the electric responses (1.5 µm of the parylene-covered delay line). The comparison of the simulated response with that measured with a VNA is presented in Figure 5. The SAW velocity *v* and attenuation $\gamma_{p}$ values cited in Table 4 are taken directly from the LDV measurements (Table 2 and Table 3) and used in Formula (1) for the modified propagation factor.

The $\kappa_{p}$ value for the non-covered sensor has been obtained by numerical simulation using FEMSDA (free software) [48,49]. This value has been used as an initial guess in the fitting procedure for the resting-covered sensors. Indeed, for a SAW delay line manufactured on a weak piezoelectric material, such as quartz, the ripples in the admittance curves are almost directly influenced by $\kappa_{p}$ [31,50]. These ripples in the conductance curve are referred to as ΔG in Figure 5b. Achieving the same amplitude of ΔG for measured and simulated curves allows a reliable extraction of the reflection coefficient value.

The transduction coefficient for the non-covered sensor, $\alpha_{p}$ has been derived from value of the effective electromechanical coupling coefficient $K^{2}$ numerically obtained with FEMSDA [31,50]:

(4) $$\alpha_{p}=\eta \sqrt{K^{2}\omega_{0}C_{n}}\sqrt{W/\lambda_{0},}$$

where η is the IDT element factor, which is equal to 0.7361 for split electrodes and 50% metallization ratio [31,51].

(5) $$C_{n}=2\cdot 0.7071\left(\varepsilon_{0}+\varepsilon_{p}^{T}\right),$$

where $\varepsilon_{p}^{T}=\varepsilon_{0}\varepsilon_{p}$ is the zero-stress permittivity, and dielectric constant $\varepsilon_{p}$ is equal to 5.506 for AT-quartz [50]. This gives 13.2 pF for the total capacitance of the non-covered IDT. According to the LDV measurements (Figure 4d), the transduction coefficient has not been impacted by the presence of parylene thin film and rests almost the same for the four considered sensors.

Modification of IDT finger resistance $R_{s}$ and capacitance C${}_{n}$ of a SAW delay line causes mainly the DC shift of the admittance curves [52]. Thus, the $C_{n}$ values for the non-covered sensors were obtained by fitting them with the VNA data. The resistance of an IDT consists of the finger resistance, the resistance of the bus bar electrode, and the contact resistance. These effects cannot be decoupled easily from a single admittance measurement. For low resistance values, it is possible to model these effects with a simple series resistor $R_{s}$ connected to the IDT [47]. Thus, the corresponding values present in Table 4 were extracted from VNA measurement data by a simple fitting procedure with the least square method.

Finally, good agreement between the simulation and measurement data was observed (Figure 5). Contrary to the initial assumptions, the reflection coefficient is affected by the parylene coating thickness. Still, its influence on the insertion loss curve is negligible in the considered split electrode configuration. Some discrepancy with impedance measurement data obtained in Section 3 is observed. According to the VNA measurements, the $C_{n}$ value increases by 9.57% when comparing the 2 µm coating and the non-covered state. Indeed, for this case, $C_{n1}$ = 0.78 pF/µm, which exceeds considerably the value of 0.15 pF/µm predicted by the simplified Equation (3).

## 7. Insight into Crosstalk Decrease

The electrical measurements have detected a decrease in crosstalk attaining −18 dB for 2 µm polymer-covered sensor (Section 3). In our previous work [35], we showed for the non-covered sensor that the crosstalk is due to the reflections on the edges of the tilted quartz substrate. This substrate tilt prevents parasitic echoes in the same channel, but on the counterpart, it makes SAW from one channel enter the substrate region occupied by the second channel. In previous work [35], additional details on the operation of the SAW device including electrically measured crosstalk echoes have been provided.

**Figure 6:**
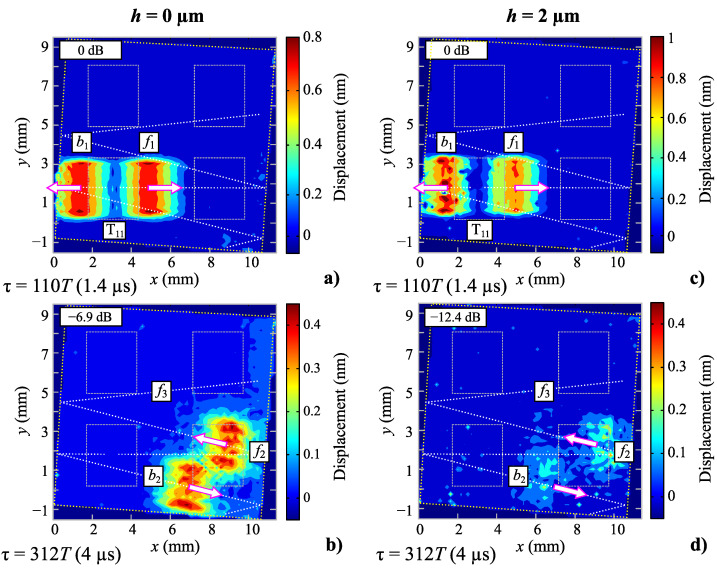
LVD C-scanning of the non-covered (**a**,**b**) and 2 µm covered (**c**,**d**) sensors.

Figure 6a,b illustrates this with LDV C-scan (left column) taken from [35]. The tilted square substrate, the IDT locations, and the SAW propagation paths are shown with white dotted lines. Figure 6a shows the time instant 1.4 µs (or 110 periods *T* of emission) shortly after the SAW emission. It can be seen later at 4 µs (312*T*) after emission that the forward propagating SAW pulse *f*${}_{1}$ (Figure 6a) undergoes a reflection from the substrate edge and becomes redirected toward the adjacent channel (*f*${}_{2}$ in Figure 6b). It must undergo a second reflection to fully enter the second channel’s region (*f*${}_{3}$ in Figure 6b, the SAW pulse is not shown). The backward-emitted SAW pulse *b*${}_{1}$ (Figure 6a) is redirected in the direction opposite to the second channel location. It does not play any significant role in the crosstalk between the two channels. Each reflection attenuates the SAW by approximately −7 dB, so the total crosstalk value is −15.2 dB. A video file for the corresponding C-scan could be found in Supplementary Material Video S1.

The same C-scanning was performed for the three sensors coated with parylene; Figure 6c,d shows the result for the case of the 2 µm coating (right column). A video file for this C-scan could be found in Supplementary Material Video S2. The same time instants (1.4 and 4 µs) are considered. It can be seen that the SAW propagation paths are unchanged, and the SAW profiles are slightly perturbed, possibly due to surface irregularities. The presence of parylene coating increases the SAW energy loss while the reflections from the substrate borders are as low as −12.4 dB. Combined with the slightly increased propagation loss, the total crosstalk value is reduced to −33.4 dB. Because of the impedance matching that the parylene thin-film layer can provide [53], such a reduction in the border reflection coefficient could be possibly caused either by the SAW radiation in the coating or SAW redirection downward in the substrate or SAW conversion in bulk waves. For this reason, a parylene C coating could reduce reflections at the edges and, thus, avoid the use of additional acoustic absorbers. Finally, it is worth mentioning that it is not straightforward to model the presented crosstalk behavior in the COM formalism, which inherently considers a 1D-SAW geometry.

## 8. Discussion

This work presents experimental data based on the electrical and optical characterization of SAW sensors coated with parylene C layers of varying thicknesses. Naturally, the effect of the polymer coating presence on SAW delay lines increases with the thickness of the coating layer. Nevertheless, for the thickest coating of 2 µm used in this study, the additional insertion loss is 3.7 dB, the frequency shift is −3.28% (2.59 MHz), and the change in static capacitance is +9.57% (1.2 pF). The measured values allowed a correct simulation of the electrical response of a coated SAW delay line using the COM theory formalism. A detailed analysis of the impact that a polymer coating has on each device parameter is presented below.

### 8.1. Frequency Shift and Sound Velocity Modification

**Figure 7:**
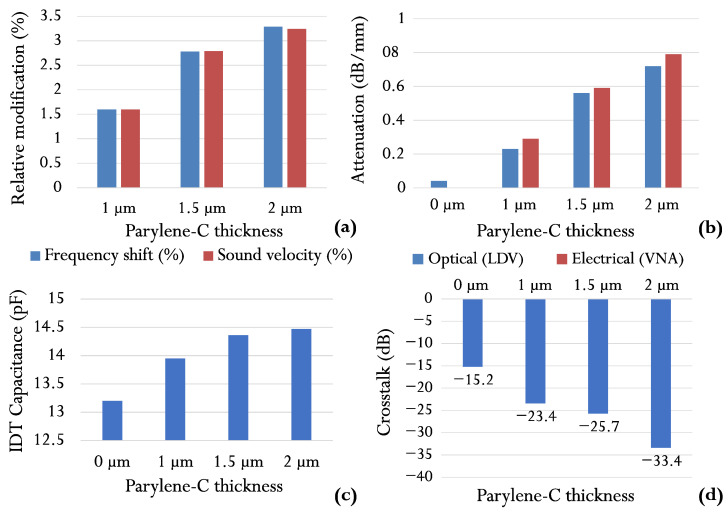
Various sensor characteristics vs. parylene coating thickness: frequency shift and sound velocity variation (**a**); attenuation (**b**); IDT capacitance (**c**); crosstalk (**d**).

The main and apparent reason for the observed frequency shift is the change in SAW velocity due to mass loading by the coating parylene layer. The electrical and optical measurements show a good agreement, especially for the uncovered SAW sensor and the SAW sensor coated with 1 µm thick layer (Table 3 and Figure 7a). A discrepancy within the limits of 0.02% (30 kHz) is observed for the cases with 1.5 µm and 2 µm coating layers, possibly due to the increased value of the additional static capacitance C${}_{p}$. Similar frequency deviation values (due to C${}_{p}$ variation) were observed in [38].

Herein, we observed that optical measurements could show the difference between the SAW velocities in the electrode grating region and the non-metalized region. This difference increases gradually with coating thickness (∼0.1% for the 2 µm coating, Table 3), revealing that the effects of the electrodes’ mass and electric loading and the coating’s mass loading are cumulative. The sound speed in the non-metalized zone affects the group delay value, and these sound velocity data are useful for fitting and validating numerical simulations.

### 8.2. Insertion and Propagation Loss

The electrical and optical methods provide a correct measurement of propagation/insertion loss due to polymer coating (Figure 7b and Table 2). The scatter between the two methods averages 0.05 dB/mm. Even for the relatively thick layer of 2 µm, the propagation loss of 0.79 dB/mm at 76.24 MHz is not too high to hinder the operation of the sensor [21]. The agreement of the results of the two methods indirectly confirms the assumption that the only mechanism leading to an increase in the losses is the absorption of the surface acoustic wave in the polymer layer and that, therefore, the efficiency of the piezoelectric conversion remains unaffected. This statement is confirmed by the optical measurement at the boundary of the emitting IDT (Figure 4d). As expected, the electrical measurements provide systematically higher values than the optical measurements, which can be explained by the influence of the peripheral components of the sensor’s packaging.

### 8.3. Static Capacity Modification

Static capacitance per period $C_{p}$ appears to have a secondary effect on IDT operation when considered as a single unit. However, when considering an oscillator measurement circuit as a whole, it is necessary to recognize that not only the SAW velocity is perturbed, but also the entire oscillator circuit is altered to some degree when $C_{p}$ is varied by the addition of a dielectric medium across the transducer fingers [38]. The total IDT capacitance *C* for each sensor is shown in Figure 7c. The extra capacity’s value increases as the sensor coating’s thickness increases. The simplified Formula (2) for $C_{n1}$ underestimates this value for all the coating thicknesses considered. For a maximum layer thickness of 2 µm, the relative increase in capacitance is 9.57%.

### 8.4. Crosstalk Decrease

Figure 7d shows the results of crosstalk measurements with the VNA. The crosstalk value decreases progressively from −15.2 to −33.4 dB for uncoated and 2 µm coated sensors. The extended dynamic range of the VNA, which is inherent to this type of instrument, allows direct and precise measurement. On the other hand, the optical LDV measurement provides insight into the reasons for such a modification, which consists of modifying the reflection coefficient from the edge of the substrate. The noise level for the LDV used is 20 pm [35]. With an initial displacement of ∼1 nm near the transmitter (Figure 4d), the displacement level in the adjacent channel is 170 pm for the uncoated sensor SAW [35] and ∼20 pm for the 2 µm coated sensor. Therefore, the dynamic range of the LDV is insufficient to quantify the latter sensor’s crosstalk value. Nevertheless, both methods are complementary in terms of the information they can provide.

## 9. Conclusions

In the final stage of the work, it was confirmed that the values found for the attenuation and the speed of sound can be used in an accurate SAW device modeling with the COM theory. The presented approach is useful for the validation of physical parameters and COM parameter fitting in the development of sensors with a biocompatible polymer thin film coating layer.

Additional robust film modeling needs to be included in the COM model considerations to account for the absorption of the measured gaseous material. Further experimental work is required before the relative contributions of dielectric, bulk, and viscoelastic effects to the response of the SAW sensor can be adequately evaluated.

In summary, we showed that the combination of electrical measurements with a vector network analyzer, optical measurements with a laser Doppler vibrometer, and numerical simulations with the finite element method coupled with the spectral domain analysis are efficient for the analysis of parylene C-coated SAW sensors. The numerical and experimental results agree and confirm that the analysis and modeling approaches are reliable for predicting the performances of the SAW sensors under surface loading.

## Figures and Tables

**Table 1:** Electrical measurements of insertion loss and central frequency.

Sensor	Central	Relative	Insertion	Increase of the Insertion	Crosstalk
	Frequency (MHz)	Downshift (%)	Loss (dB)	Loss Value (dB)	(dB)
Uncoated	78.83	n/a	−29.95	n/a	−15.2
Coated					
1 µm	77.55	1.6	−31.23	1.28	−23.4
1.5 µm	76.64	2.7	−32.83	2.88	−25.7
2 µm	76.24	3.28	−33.85	3.9	−33.4

**Table 2:** Comparison of optical and RF loss measurements as functions of parylene thickness.

Parylene Thickness (µm)	Optical Loss (dB/mm)	RF Loss (dB/mm)	Difference
0	0.04	...	...
1 µm	0.23	0.29	0.06
1.5 µm	0.56	0.59	0.03
2 µm	0.72	0.79	0.07

**Table 3:** VNA measurements of central frequency shift (first and second columns), the results of optical sound velocity measurements (third and fourth columns) and a comparison of the two measurement methods in terms of frequency (fifth and sixth columns, $\lambda_{0}$ = 40 µm).

Thickness	VNA Frequency	Relative Shift	Velocity, IDT Free	Velocity, IDT	LDV Frequency	Relative Shift
(µm)	(MHz)	%	Space (m/s)	Region (m/s)	(MHz)	%
0 µm	78.83	–	3156	3153	78.83	–
1 µm	77.55	1.6	3105	3102	77.55	1.6
1.5 µm	76.64	2.7	3068	3065	76.63	2.79
2 µm	76.24	3.28	3057	3051	76.27	3.25

**Table 4:** COM parameters for split aluminum electrodes on AT-cut quartz substrate for different parylene C coating thicknesses ($h⁄\lambda_{0}=0.5\%$).

Parameters	0 µm	1 µm	1.5 µm	2 µm
υ (m/s)	3153	3101	3065	3051
$\kappa_{p}$ (%)	0.371	0.239	0.17	0.145
$\alpha_{n}\left(\Omega^{-1/2}/\sqrt{\frac{W}{\lambda_{0}}}\right)$	2.24 × 10${}^{-5}$	2.218 × 10${}^{-5}$	2.18 × 10${}^{-5}$	2.216 × 10${}^{-5}$
C${}_{n}$ (pF/µm)	8.15 × 10${}^{-5}$	8.67 × 10${}^{-5}$	8.87 × 10${}^{-5}$	8.93 × 10${}^{-5}$
$\gamma_{p}\left(N_{p}/\lambda_{0}\right)$	1.84 × 10${}^{-4}$	1.059 × 10${}^{-3}$	2.58 × 10${}^{-3}$	3.32 × 10${}^{-3}$
$R_{s}$ (Ohm)	n/a	11.55	11.25	12.2

## Data Availability

Not applicable.

## Supplementary Materials

The following supporting information can be downloaded at: https://www.mdpi.com/article/10.3390/s22228611/s1, Video S1: LDV C-scanning of the non-covered sensor. Video S2: LDV C-scanning of the 2 µm Parylene-covered sensor.

## Abbreviations

The following abbreviations are used in this manuscript:

SAW	surface acoustic wave
COM	coupling-of-modes
VNA	vector network analyzer
LDV	laser Doppler vibrometry
FEMSDA	finite element method and spectral domain analysis
